# Meteorological Report

**Published:** 1879-12

**Authors:** H. Hall

**Affiliations:** Corp’l. Signal Service U.S. A.; Atlanta, Ga.


### METEOROLOGICAL REPORT FOR NOVEMBER, 1879.

| RAIN. DATE | IN. | Mean daily Barometer. | Mean daily Thermometer | Mean daily Humidity. | Maximum Thermometer | Minimum Thermometer | Prevailing direction of Wind. | Maximum hourly velocity of Wind, (Daily.) |
|---|---|---|---|---|---|---|---|---|
| 1 | 0 | 30.268 | 52.7 | 57.0 | 58 | 46 | ......... | ......... |
| 2 | 0 | 30.148 | 53.7 | 53.3 | 63 | 47 | ......... | ......... |
| 3 | 0 | 30.239 | 41.2 | 56.3 | 52 | 36 | ......... | ......... |
| 4 | 0 | 30.350 | 41.7 | 53.3 | 50 | 30 | ......... | ......... |
| 5 | 0 | 30.381 | 45.5 | 46.7 | 52 | 37 | ......... | ......... |
| 6 | 0 | 30.219 | 56.5 | 68.0 | 66 | 39 | ......... | ......... |
| 7 | 0 | 30.139 | 61.2 | 81.7 | 68 | 50 | ......... | ......... |
| 8 | 05 | 30.130 | 64.2 | 88.3 | 69 | 59 | ......... | ......... |
| 9 | 04 | 30.219 | 67.2 | 89.3 | 71 | 63 | ......... | ......... |
| 10 | 09 | 30 258 | 67.0 | 90.7 | 70 | 64 | ......... | ......... |
| 11 | 02 | 30.167 | 66.7 | 84.3 | 73 | 61 | ......... | ......... |
| 12 | 0 | 30.076 | 68.0 | 82.0 | 75 | 60 | ......... | ......... |
| 13 | 0 | 30.088 | 70.5 | 85.0 | 75 | 66 | ......... | ......... |
| 14 | 0 | 29.951 | 70.0 | 74.3 | 75 | 65 | ......... | ......... |
| 15 | 43 | 29.852 | 69.2 | 85.3 | 76 | 64 | ......... | ......... |
| 16 | 0 | 30.063 | 63.5 | 70.0 | 71 | 59 | ......... | ......... |
| 17 | 0 | 30.059 | 54.2 | 82.0 | 59 | 52 | ......... | ......... |
| 18 | 29 | 30 037 | 47.0 | 88.0 | 59 | 41 | ......... | ......... |
| 19 | 1.19 | 30.022 | 41.2 | 85.3 | 48 | 35 | ......... | ......... |
| 20 | 04 | 30 206 | 34.2 | 41.7 | 42 | 29 | ......... | ......... |
| 21 | 0 | 30.273 | 34 0 | 50.7 | 44 | 24 | ......... | ......... |
| 22 | 0 | 30.214 | 44.0 | 49.7 | 51 | 31 | ......... | ......... |
| 23 | 0 | 30.204 | 49.7 | 44.3 | 60 | 37 | ......... | ......... |
| 24 | 0 | 30.334 | 46.7 | 43.7 | 56 | 35 | ......... | ......... |
| 25 | 0 | 30.304 | 53.7 | 55.0 | 60 | 40 | ......... | ......... |
| 26 | 0 | 30.314 | 57.0 | 49.7 | 65 | 49 | ......... | ......... |
| 27 | 10 | 30.190 | 56.0 | 84.0 | 60 | 41 | ......... | ......... |
| 28 | 1.63 | 29.930 | 59.7 | 93.7 | 62 | 57 | ......... | ......... |
| 29 | 0 | 30.255 | 41.5 | 53.0 | 60 | 38 | ......... | ......... |
| 30 | 0 | 30.434 | 39.0 | 46.3 | 46 | 32 | ......... | ......... |
| 31 | .. | ......... | ...... | ...... | ... | ... | ......... | ......... |
| Sums | 3.88 | 905.314 | 15.167 | 20.426 | 18.36 | 13.86 | ......... | ......... |

Highest Barometer, 30.452 on the 30th.
Lowest Barometer, 29.762, on the 15th.
Monthly range of Barometer, 0.690.
Highest Temp. 76°, on 15th. Lowest Temp. 24°, on 21st.
Monthly range of Temperature, 52°.
Greatest daily range of Temperature, 27°, on the 6th.
Lowest daily range of Temperature, 5°, on the 28th.
Mean of Maximum Temperatures, 61.2.
Mean of Minimum Temperatures, 46.2.
Mean daily range of Temperature, 15.0. Total rainfall, 3.88 inches.
Prevailing wind, S. E. Total movement of wind, 72.52 miles.
Maximum velocity of wind and direction, 36 miles per hour, N. W., 20th.
Number of Foggy days, 0.
Number Cloudy days on which rain fell, 9.
Number of cloudy days on which no rain fell, 3.
Total number of days on which rain fell, 12.

H. HALL, Corp'l. Signal Service U. S. A.

Atlanta, Ga., Dec. 1, 1879.

